# Testing the effects of explicit and implicit bidimensional attitudes on objectively measured speeding behaviour

**DOI:** 10.1111/bjso.12255

**Published:** 2018-03-30

**Authors:** Rebecca McCartan, Mark A. Elliott, Stefania Pagani, Eimear Finnegan, Steve W. Kelly

**Affiliations:** ^1^ School of Psychological Sciences and Health University of Strathclyde Glasgow UK

**Keywords:** bidimensional attitudes, explicit attitudes, implicit attitudes, speeding behaviour

## Abstract

Bidimensional attitudes have been shown to independently predict behaviour, with the positive dimension of attitude being a stronger predictor of behaviour than the negative dimension (e.g., Elliott, Brewster, *et al*., 2015, *Br. J. Psychol, 106*, 656). However, this positivity bias has been demonstrated with explicit attitude measures only and explicit attitude measures tap deliberative processes rather than automatic processes, which are known to be important in the execution of many behaviours. The aim of this study was to test whether implicit bidimensional attitudes can account for variance in speeding behaviour over and above explicit bidimensional attitudes and whether the positivity bias that is typically found with explicit attitudes generalizes to implicit attitudes. A total of 131 drivers completed a questionnaire measuring their explicit bidimensional attitudes towards speeding. They also completed Implicit Association Tests measuring their implicit bidimensional attitudes. Two weeks later, speeding behaviour was measured using a driving simulator. Explicit attitudes accounted for a significant proportion of the variance in subsequent speeding behaviour. Implicit attitudes accounted for a statistically significant increment to explained variance. The positive dimension of both explicit and implicit attitudes predicted speeding behaviour but the negative dimensions did not. Theoretical implications for understanding the potential attitudinal causes of behaviour and practical implications for behaviour‐change interventions are discussed.

## Background

Attitudes are typically treated as unidimensional predictors of behavioural intentions and subsequent behaviour (e.g., Armitage & Conner, 2001; Eagly & Chaiken, 1993). In line with traditional conceptualizations of the attitude construct (e.g., Osgood, Suci, & Tannenbaum, 1957; Thurstone, 1928), this means that individuals are held to evaluate behaviours along a single, bipolar, positive–negative dimension (e.g., ‘For me, speeding is negative or positive’). The likelihood of a behaviour being performed is then held to increase with the extent to which it is evaluated positively rather than negatively (e.g., Fishbein, 1963). More recently, however, attitudes have been conceptualized as bidimensional predictors of behaviour (Elliott, Brewster, Thomson, Malcolm, & Rasmussen, 2015; McCartan & Elliott, 2018). This means that attitudes comprise separate unipolar, positive, and negative dimensions (e.g., ‘For me, speeding is not at all positive to extremely positive’ and ‘For me, speeding is not at all negative to extremely negative’). The likelihood of a behaviour being performed is then held to increase with the extent to which it is evaluated positively and, at the same time, the extent to which it is not evaluated negatively. In this article, we present a study in which we tested these effects of bidimensional attitudes on drivers’ speeding behaviour. In line with recent research, we aimed to test the effects of explicit, questionnaire‐based measures of bidimensional attitudes on subsequent behaviour. We also aimed to extend previous research by testing the extent to which implicit, cognitive test‐based measures of bidimensional attitudes could increase the prediction of subsequent behaviour over and above the explicit measures.

Traditionally, attitudes have been conceptualized as unidimensional evaluations that are measured on single, bipolar scales (Allport, 1935). Several researchers, however, have questioned the singularity of the attitude construct and have distinguished between different components of attitudes. In particular, it is common for researchers (e.g., Elliott & Thomson, 2010; Elliott, Thomson, Robertson, Stephenson, & Wicks, 2013; Lawton, Conner, & McEachan, 2009; Rhodes, Blanchard, & Matheson, 2006) to distinguish between cognitive attitudes (positive or negative instrumental evaluations; e.g., ‘For me, speeding is harmful/beneficial’) and affective attitudes (e.g., positive or negative emotional evaluations; e.g., ‘For me, speeding is enjoyable/unenjoyable’). In both cases, however, attitudes are treated as unidimensional (i.e., positive or negative). This unidimensionality has previously attracted criticism (e.g., Kaplan, 1972) because it makes the midpoint of an attitude scale ambiguous, indicating either attitudinal indifference (i.e., a state that occurs when a behaviour is simultaneously evaluated as neither positive nor negative) or attitudinal ambivalence (i.e., a state that occurs when a behaviour is simultaneously evaluated as both positive and negative). As a solution to this problem, Kaplan (1972) recommended splitting the single positive/negative attitude dimension at its midpoint, thus producing a positive dimension and a separate negative dimension (i.e., bidimensional attitudes). Operationally, Kaplan (1972) recommended the split semantic differential technique as a method for measuring the two attitude dimensions because it allows individuals to evaluate the positive and negative attributes (consequences) of a behaviour independently on separate (positive and negative) unipolar scales, thereby acknowledging the possibility that positive and negative attitudes towards the same behaviour can coexist (Thompson, Zanna, & Griffin, 1995).

In support of the bidimensional conceptualization of an attitude, factor analytic studies have demonstrated that positive and negative behavioural evaluations load separately onto two independent dimensions (e.g., Conner *et al*., 2002). However, attitudes have continued to be treated as unidimensional constructs when testing the relationships between attitudes, on the one hand, and measures of behavioural intentions or subsequent behaviour, on the other hand (for reviews, see McEachan, Conner, Taylor, & Lawton, 2011; Wallace, Paulson, Lord, & Bond, 2005). Even in studies of attitudinal ambivalence (for a review, see Conner & Sparks, 2002), a primary focus has been to demonstrate that evaluative conflict between the separate positive and negative attitude dimensions (i.e., attitude ambivalence) moderates the relationship between overall (i.e., unidimensional) measures of attitudes, on the one hand, and measures of behavioural intentions or subsequent behaviour, on the other hand, with greater evaluative conflict leading to poorer attitude–behaviour relationships (see Conner & Sparks, 2002). The rationale is that the evaluative conflict, which stems from simultaneously evaluating a behaviour as both positive and negative, is indicative of weak attitudes, which are poor predictors of behaviour (Glasman & Albarracín, 2006; Kraus, 1995).

Notwithstanding the importance of research on attitudinal ambivalence, a serious acceptance of the bidimensional conceptualization of attitudes requires that the positive and negative dimensions are treated as independent predictors of behaviour. While it is conceptually redundant to treat attitude ambivalence as a moderator of these relationships (i.e., it is circular to reason that a measure based on two independent constructs should moderate the effects of those same constructs on an outcome),^1^ treating the separate positive and negative attitude dimensions as independent predictors of behaviour is important nonetheless. It allows researchers to test potential differences between their predictive validities, which has important implications for better understanding behaviour (i.e., which attitude dimension is the better predictor of behaviour?) and the development of effective interventions (i.e., which attitude dimension might need prioritizing in behaviour‐change efforts?).

Recent research in which attitudes have been treated as bidimensional predictors of behaviour (Elliott, Brewster, *et al*., 2015; McCartan & Elliott, 2018) has shown that both the positive and negative attitude dimensions independently predict binge‐drinking intentions (Elliott, Brewster, *et al*., 2015 [studies 1 and 2]), smoking and unhealthy dieting intentions (Elliott, Brewster, *et al*., 2015 [study 2]), self‐reported speeding behaviour (Elliott, Brewster, *et al*., 2015 [study 3]), and objectivity measured speeding behaviour (McCartan & Elliott, 2018). These studies have also shown that the positive dimension of attitude is a significantly stronger predictor of both intentions and subsequent behaviour than is the negative dimension. This finding is in line with the majority of previous studies on expectancy beliefs (precursors to attitudes) in which beliefs about the likelihood of positive behavioural outcomes have been found to predict behaviour to a greater extent than beliefs about the likelihood of negative behavioural outcomes. For example, Lee, Greely, and Oei (1999) found that positive expectancy beliefs accounted for more variance in binge‐drinking behaviour than did negative expectancy beliefs. Lawton, Conner, and Parker (2007 [study 2]) found that positive expectancy beliefs had larger standardized regression weights than did negative expectancy beliefs in the prediction of smoking behaviour (also see Anderson, Pollak, & Wetter, 2002). Similarly, Rhodes and Conner (2010) found that positive expectancy beliefs had larger standardized regression weights than did negative expectancy beliefs in the prediction of physical activity intentions, in particular for affective rather than cognitive and proximal rather than distal outcomes. Furthermore, Fromme, Katz, and Rivet (1997) found that positive expectancy beliefs had larger standardized regression weights than did negative expectancy beliefs in the prediction of a range of behaviours including drug‐use, heavy‐drinking, and engagement in illegal activities such as drink driving. Although Lawton *et al*. (2007 [study 1]) found that expectancy beliefs about negative affective outcomes had larger standardized regression weights in the prediction of speeding behaviour than did expectancy beliefs about positive affective outcomes, the findings of previous research, overall, suggest a ‘positivity bias’ in behavioural decision‐making (e.g., Boucher & Osgood, 1969). With regard to bidimensional attitudes, evaluations of positive behavioural outcomes outweigh evaluations of negative behavioural outcomes in the prediction of behaviour (but see Lawton *et al*., 2007 [study 1]).

A potential limitation of the above cited studies, however, is that they have all focused exclusively on explicit attitudes. Explicit attitudes are attitudes of which an individual is consciously aware. Consequently, they are held to influence behaviour through a deliberative process, with individuals consciously considering the positive and negative outcomes of a behaviour before engaging in it (e.g., Elliott, Lee, Robertson, & Innes, 2015; Fazio & Olson, 2003; Fazio, 1990; Spalding & Hardin, 1999). This raises two potential concerns. First, many real‐world behaviours (e.g., speeding) are readily repeatable and are therefore afforded the opportunity to become automatic. This means that spontaneous processes are likely to be involved in the execution of behaviour in addition to more deliberative processes (e.g., Verplanken & Orbell, 2003). Second, explicit attitudes are typically measured using self‐report questionnaires, which can be susceptible to various cognitive biases, such as primary and recency effects (Murdock, 1962), affective biases such as mood congruent memory effects (e.g., Mayer, McCormick, & Strong, 1995), and self‐presentation biases such as self‐deception (Gur & Sackeim, 1979) and impression management (Paulhus & Reid, 1991). Implicitly measured attitudes help to overcome these potential problems. This is because implicit attitudes are attitudes of which individuals are *not* consciously aware. Consequently, they are held to influence behaviour through a spontaneous, rather than a deliberative, process. More specifically, they are held to be activated spontaneously when individuals encounter salient cues that are associated with a behaviour. These automatically activated attitudes are then held to exert a biasing effect on an individual, effectively priming (initiating rapidly and without conscious awareness) attitude‐congruent behaviour (e.g., Fazio, 1990; Fazio & Olson, 2003). In addition, implicit attitudes are not vulnerable to self‐reporting biases because they are measured by performance on cognitive tests, rather than self‐report questionnaires (e.g., Banse, Seise, & Zerbes, 2001).

The most commonly employed method for measuring implicit attitudes is the Implicit Association Test (IAT; Greenwald, McGhee, & Schwartz, 1998; Greenwald, Nosek, & Banaji, 2003; but see Fazio, 2001 for an alternative method). The IAT is a computer‐based reaction time task that assesses the strength of associations between ‘target concepts’ (e.g., behaviours) and ‘attributes’ (e.g., evaluations). In a standard, traditional IAT, a target concept (e.g., speeding) is presented on one side of a computer screen and its opposite concept (e.g., complying) is presented on the other side. Each concept is paired with an attribute (e.g., speeding/good; complying/bad). The participants are presented with items in the middle of the screen relating to both the concepts (e.g., illegal or legal) and the attributes (e.g., happy or nasty). The participants’ task is to categorize each item into its relevant category as quickly and accurately as possible (e.g., Greenwald *et al*., 1998). A measure of attitude (e.g., towards speeding) is then derived from the difference in the participants’ response latencies (i.e., time taken to categorize items) in ‘compatible trials’, when the target concept is paired with ‘good’ and its opposite concept is paired with ‘bad’, and their response latencies in the ‘incompatible trials’, when the target concept is paired with ‘bad’ and its opposite concept is paired with ‘good’ (Greenwald *et al*., 1998, 2003). The rationale is that faster response latencies in compatible relative to incompatible trials indicate a positive attitude towards the target concept.

As an example, imagine a driver with a positive attitude towards speeding. This driver would be able to more quickly categorize items in an IAT when ‘speeding’ is paired with ‘good’ and ‘complying’ is paired with ‘bad’ (i.e., the compatible trials) than when ‘speeding’ is paired with ‘bad’ and ‘complying’ is paired with ‘good’ (i.e., the incompatible trials). This is because his or her pre‐existing cognitive association between ‘speeding’ and ‘good’ (i.e., his or her positive attitude towards speeding) is facilitating task performance in the compatible trials and inhibiting it in the incompatible trials (e.g., Greenwald *et al*., 1998). On the other hand, a driver with a negative attitude towards speeding would be able to more quickly categorize items in an IAT when ‘speeding’ is paired with ‘bad’ and ‘complying’ is paired with ‘good’ than when ‘speeding’ is paired with ‘good’ and ‘complying’ is paired with ‘bad’. This is because his or her pre‐existing cognitive association between ‘speeding’ and ‘bad’ (i.e., his or her negative attitude towards speeding) is facilitating task performance in the incompatible trials and inhibiting it in the compatible trials.

Several studies have demonstrated that implicit attitudes can predict health‐risk behaviours, such as smoking (Chassin, Presson, Sherman, Seo, & Macy, 2010), binge‐drinking (Houben, Havermans, & Wiers, 2010), and speeding (Hatfield, Fernandes, Faunce, & Job, 2008). However, these studies have all focused on unidimensional rather than bidimensional measures of attitudes, using IATs such as the one described above. Given the findings from studies of explicit bidimensional attitudes, it seems plausible that implicit attitudes will also have separate positive and negative dimensions that independently predict behaviour. One aim of this research therefore was to measure implicit bidimensional attitudes and test the extent to which they predict subsequent behaviour. We focused on drivers’ speeding behaviour because it is a behaviour that typically results in both positive and negative outcomes and therefore tends to generate both positive and negative dimensions of attitudes (e.g., Elliott, Armitage, & Baughan, 2005). Additionally, speeding is a prominent cause of road traffic accidents and casualties (Department for Transport, 2016). Research that identifies potentially modifiable predictors of speeding (e.g., attitudes) is therefore important for understanding this behaviour and finding ways to reduce it.

An additional aim of this study was to explore the relative effects of implicit and explicit attitudes in the prediction of behaviour. Understanding the interplay between deliberative and automatic decision‐making in the prediction of behaviour is particularly important for behaviours such as speeding because both types of processes are likely to influence action. For example, in certain situations (e.g., driving on a road with a speed camera), deliberative decision‐making, reflected in explicit attitudes, is likely to dictate behaviour. However, in other situations (e.g., driving on a well‐rehearsed journey in which nothing out of the ordinary occurs and does not therefore require high levels of conscious deliberation), automatic decision‐making, reflected in implicit attitudes, is likely to dictate behaviour.

Several studies have examined the effects of both implicit and explicit attitudes on behaviour (e.g., Gawronski, Galdi, & Arcuri, 2015; Greenwald, Poehlman, Uhlmann, & Banaji, 2009; Spence & Townsend, 2010). A meta‐analysis of *k *=* *152 independent studies by Greenwald *et al*. (2009) found that implicit attitudes accounted for an increment to explained variance in behaviour over and above the variance accounted for by explicit attitudes, and both types of attitude accounted for unique variance in behaviour. However, no previous studies have simultaneously tested the predictive validity of implicit and explicit attitudes on speeding and no studies have tested the effects of both implicit and explicit *bidimensional* attitudes on any behaviour. It was expected in this study that implicit bidimensional attitudes would predict speeding behaviour over and above explicit bidimensional attitudes.

In line with the above discussion, the aim of this research was to test the effects of implicit bidimensional attitudes on behaviour over and above the effects of explicit bidimensional attitudes. In line with previous research (e.g., Elliott, Brewster, *et al*., 2015; McCartan & Elliott, 2018), hypothesis 1 was that the positive and negative dimensions of explicit attitudes would account for a significant proportion of the variance in behaviour, with the positive dimension being the stronger predictor. Hypothesis 2 was that the positive and negative dimensions of implicit attitudes would account for a significant increment to explained variance in behaviour, with the positive dimension again being the stronger predictor.

## Method

### Participants

One hundred and thirty‐one active drivers (full UK driving licence holders who drove at least once a week) took part. The participants were recruited using advertisements placed on notice boards around the campus of a large university in the West of Scotland and online posts (e.g., advertisements on social networking sites and the virtual learning environment of the university). The mean age of the sample was 22.66 (*SD *= 8.50; range* *= 18–65), and 21.4% was male (*N *=* *28). The mean weekly mileage was 74.81 (*SD *= 75.90; range* *= 1–400), and the mean number of years that the participants had held a full driving licence for was 4.09 (*SD *= 6.84; range* *= 0.16–38).

Power analysis indicated that the power provided by the present sample (*n* = 131) to detect a meaningful (small to moderate) sized relationship (*r *=* *.22 for correlation and *f* ^2^ **= .10 for regression with four independent variables) was power* *= 0.82. Given the power estimate was above 0.80 (see Cohen, 1992), it was concluded that the present study was sufficiently powered for testing the hypotheses.

### Design and procedure

A prospective design was used. The participants were invited to participate after being informed the study was a general‐purpose investigation into driver behaviour and attitudes. All of the participants were invited to the social cognition laboratory situated in the School of Psychological Sciences and Health. After providing their consent, the participants completed a questionnaire that contained standard items to measure basic demographic information (e.g., age, gender, weekly mileage, number of years licensed to drive) and explicit attitudes towards exceeding the speed limit (both the positive and negative dimensions, separately). The participants also completed IATs to measure their implicit attitudes towards exceeding the speed limit (again, both the positive and negative dimensions, separately). The questionnaire took approximately 5 min to complete and was developed and administered using Qualtrics. The IATs took approximately 15 min to complete and were developed and administered using E‐Prime. Half of the participants received the questionnaire first and half received the IATs first to control for any potential order effects.

Two weeks later, the participants were invited to the Driving Research Laboratory situated in the School of Psychological Sciences and Health where objective measures of speeding were obtained from a driving simulator. After completing the simulator drive, the participants were thanked and debriefed.

### The explicit attitude measures

Explicit attitudes were measured using standard questionnaire items (i.e., commonly employed in the literature and shown to procedure reliable measures). The participants were asked to respond to the items that measured their attitudes towards exceeding the speed limit using 9‐point scales. All attitude items were presented in a pseudo‐random order, with the response scales reversed for half the items to reduce response set bias (e.g., Nederhof, 1985). These measures were presented amongst ‘filler items’ asking the participants about their general driving practices (e.g., how often they drive in urban areas) to help prevent consistency biases (e.g., Budd, 1987) from influencing the participants’ responses.

The split semantic differential technique (Kaplan, 1972) was used to measure the separate positive and negative dimensions of attitude. Four items were used to measure the positive dimension. The participants were asked to ‘Think only about the positive/rewarding/beneficial/pleasant outcomes that you associate with speeding’ and to rate ‘How positive/rewarding/beneficial/pleasant are they?’ The participants’ ratings were provided on scales from ‘not at all positive/rewarding/beneficial/pleasant’ (scored 1) to ‘extremely positive/rewarding/beneficial/pleasant’ (scored 9). The mean of the four items was calculated and used as the final measure of the explicit positive attitude dimension. The Cronbach's alpha was α = .88 and therefore was judged to possess internal reliability (i.e., α > .70; Cronbach, 1951; Nunnaly, 1978). Higher scores indicated that the positive outcomes of speeding were rated more positively.

Four items were also used to measure the negative dimension of attitude. The participants were asked to ‘Think only about the negative/unrewarding/harmful/unpleasant outcomes that you associate with speeding’ and to rate ‘How negative/unrewarding/harmful/unpleasant are they?’ The ratings were provided on a scale from ‘not at all negative/unrewarding/harmful/unpleasant’ (scored 1) to ‘extremely negative/unrewarding/harmful/unpleasant’ (scored 9). The mean of the four items provided a reliable final measure of the explicit negative attitude dimension (Cronbach's α = .73). Higher scores indicated that the negative outcomes of speeding were rated more negatively.

### The implicit attitude measures

A standard IAT, as described in the Introduction, is appropriate for measuring implicit unidimensional attitudes (i.e., positive or negative associations). However, ‘single‐attribute’ IATs (e.g., Penke, Eichstaedt, & Asendorpf, 2006) are required to measure the separate positive and negative dimensions of implicit bidimensional attitudes. Single‐attribute IATs are typically used when an attribute has no clear opposite category (e.g., sociosexuality: Penke *et al*., 2006). However, single‐attribute IATs have not previously been used to measure bidimensional attitudes. Single‐attribute IATs were therefore developed to measure implicit bidimensional attitudes in this research.

The single‐attribute IAT to measure the positive dimension of attitude comprised five blocks of ‘trials’ (see Table 1). In block 1, the participants were shown a screen. The target‐concept category ‘speeding’ was presented at the top of one side of the screen. The opposite‐concept category ‘complying’ was presented at the top of the other side of the screen. The participants were then shown a series of items in the centre of the screen that either belonged to the ‘speeding’ or ‘complying’ concept categories. The participants were asked to correctly categorize these items as quickly and accurately as possible. The participants were presented with five items related to ‘speeding’ (fast, rush, speeding, illegal, and disobey) and five items related to ‘complying’ (slow, cautious, adhere, legal, and comply). Each item was presented twice, meaning that there were 20 trials in total. The participants were asked to press the ‘E’ key on the computer keyboard when item belonged to the concept category on the left of the screen and to press the ‘I’ key when the item belonged to the category on the right. The items remained on the screen until a response was given and, if an incorrect response was given (e.g., if a speeding‐related item was categorized as ‘complying’), an X appeared in the centre of the screen until the correct response was provided.

**Table 1:** Sequence of trial blocks for single‐attribute Implicit Association Test (IAT) measuring the positive dimension of attitude

Block	No. of trials (i.e., words per block)	Function	Top left of screen in version 1 of the IAT	Top right of screen in version 1 of the IAT
1	20	Practice	Speeding	Complying
2	20	Practice	Speeding + Good	Complying
3	40	Test	Speeding + Good	Complying
4	20	Practice	Speeding	Complying + Good
5	40	Test	Speeding	Complying + Good

In block 2, the participants were presented with the same display as in block 1, except that the single‐attribute category ‘good’ was paired with the concept category at the top left of the screen. The participants were then presented with the same series of ‘speeding’/’complying’ items used in block 1. They were also presented with five items related to the attribute category ‘good’ (happy, fun, wonderful, positive, and enjoyable). Each of the items from the concept and attribute categories (‘speeding’, ‘complying’, and ‘good’) was presented at least once. Five of the items were shown twice. Of these items, three items belonged to the concept category on the left side of the screen, one belonged to the concept category on the right, and one belonged to the attribute category. This meant that block 2 comprised 20 trials and the number of items that the participants needed to categorize on the left and right sides of the screen was proportional to the number of categories. As in block 1, the participants were asked to press ‘E’ on the keyboard when the item belonged a category on the left of the screen and ‘I’ when it belonged to the category on the right.

In block 3, the participants completed the same task as in block 2 except that they needed to classify twice as many items (i.e., there were 40 trials in block 3). In blocks 4 and 5, the participants were given the same tasks as in blocks 2 and 3 except that the attribute category ‘good’ was paired with the concept category on the right rather than the left of the screen.

There were two different versions of this single‐target IAT. In version 1, the target‐concept category ‘speeding’ was presented on the left‐hand side of the screen and the opposite‐concept category ‘complying’ was on the right. In version 2, this was reversed. Half of the participants were selected at random to receive version 1, and half were selected at random to receive version 2 to counterbalance across the sample. This procedure was used to address the commonly found order effect in IAT research, with performance on the compatible or incompatible trials being faster when it is completed first (i.e., blocks 2 and 3) compared with when it is completed second (i.e., blocks 4 and 5; e.g., Greenwald *et al*., 2003). In both IATs, the intertrial interval (milliseconds between each trial) used was 250 ms, consistent with standard practice (e.g., Greenwald *et al*., 2003).

Regardless of which version of the IAT the participants received, the response latencies to the blocks in which the attribute category was paired with the ‘speeding’ target‐concept category (commonly referred to as ‘compatible trials’) and the blocks in which it was paired with the ‘complying’ opposite‐concept category (commonly referred to as ‘incompatible trials’) were used to derive a ‘*D*‐score’ (see Greenwald *et al*., 2003). This *D*‐score served as the implicit measure of the positive attitude dimension. Following Greenwald *et al*. (2003), the mean response latencies (the time in milliseconds it took for participants to correctly classify each item) for the compatible trials were subtracted from the mean response latencies for the incompatible trials, meaning that higher scores equated to faster categorization of items when ‘good’ was paired with ‘speeding’ rather than ‘complying’ (i.e., higher scores equated to more positive attitudes towards speeding). The difference between the participants’ mean latencies of response in the compatible versus incompatible trials was divided by the standard deviation across the compatible and incompatible trials to produce an overall measure of effect size (i.e., *D*). In line with standard practice (i.e., Greenwald *et al*., 2003), this procedure was used to calculate a *D*‐score for blocks 2 versus 4 and 3 versus 5 separately and the mean of the two scores served as the final measure (*D*) of the positive dimension of the participants’ implicit attitudes towards speeding.^2^ The Cronbach's alphas for versions 1 and 2 of this IAT were α = .72 and α = .66, respectively. This meant that the Cronbach's alpha for the overall measure of the positive dimension of implicit attitudes was α = .69.

The negative dimension of implicit attitudes was also measured using a single‐attribute IAT (see Table 2). This IAT was the same as the one used to measure the positive dimension of implicit attitudes except that the attribute category ‘bad’, rather than ‘good’, was paired with the target‐concept category ‘speeding’ and the opposite‐concept category ‘complying’ in blocks 2–5. In addition to correctly categorizing the items relating to speeding and complying, the participants therefore had to correctly categorize items relating to the attribute category ‘bad’ (evil, disaster, awful, negative and nasty). Once again, there were two different versions of the IAT. In version 1, the opposite‐concept category ‘complying’ was presented on the left‐hand side of the screen and the target‐concept category ‘speeding’ was presented on the right. In version 2, this was reversed. To counterbalance across the sample, the participants who received version 1 of the IAT to measure the positive dimension of attitude received version 1 of this IAT. Similarly, the participants who received version 2 of the IAT to measure the positive dimension of attitude received version 2 of this IAT. To control for any potential practice effects, the order in which the participants received their two IATs varied. The participants either received the positive single‐attribute IAT first or the negative single‐attribute IAT first.

**Table 2:** Sequence of trial blocks for single‐attribute Implicit Association Test (IAT) measuring the negative dimension of attitude

Block	No. of trials (i.e., words per block)	Function	Top left of screen in version 1 of the IAT	Top right of screen in version 1 of the IAT
1	20	Practice	Complying	Speeding
2	20	Practice	Complying + Bad	Speeding
3	40	Test	Complying + Bad	Speeding
4	20	Practice	Complying	Speeding + Bad
5	40	Test	Complying	Speeding + Bad

A final measure of the negative dimension of the participants’ implicit attitudes towards speeding was calculated following the same procedure as for the positive dimension of their implicit attitudes. The mean of the response latencies in the incompatible trials (‘bad’ paired with ‘complying’) was subtracted from the mean of the response latencies in the compatible trials (‘bad’ paired with ‘speeding’), meaning that higher scores reflected more negative attitudes towards speeding. This difference was then divided by the standard deviation across the compatible and incompatible trials for the ‘practice’ and ‘test’ blocks separately and the mean of the resulting *D*s served as the final measure (*D*) of the negative dimension of the participants’ implicit attitudes. The Cronbach's alphas for versions 1 and 2 of this IAT were α = .61 and α = .68, respectively. This meant that the Cronbach's alpha for the overall measure of the negative dimension of implicit attitudes was α = .64).

It should be noted that the data from all IATs were trimmed following standard procedures (Greenwald *et al*., 2003). All response latencies that were over 10,000 ms were removed to ensure that the data were not contaminated by trials that were ‘abnormally slow’. Across all IATs, there were just *n *=* *5 participants with response latencies over 10,000 ms. In each case, the maximum number of abnormally slow trials was just *n *=* *2. These participants’ final *D* scores were calculated using the remaining latencies. Greenwald *et al*. (2003) also recommend that participants should be removed from the sample if more than 10% of their response latencies are <300 ms to prevent contamination by ‘abnormally fast’ trials. In this study, there were no participants with more than 10% of their trials <300 ms.

### The speeding behaviour measure

The driving simulator used in this study was an interactive fixed‐based driving simulator modelled on the layout of a British car (i.e., right‐hand drive). The simulator had three high‐resolution screens to the front, providing 210 degree visual field of view. The simulator operated with an automatic transmission and had controls (e.g., a steering wheel, indicators, clutch, brake, and accelerator) that are situated and operate as in a real‐world vehicle. The rear‐view mirror was shown at the top of the centre screen, and a speedometer and tachometer were shown at the bottom. The wing mirrors were shown on the side screens.

The participants were initially given a 5‐min practice drive to get used to the simulator controls. Following the practice drive, the participants completed the trial route, which comprised a 7.06‐mile section of road through an urban environment, taking approximately 15 min to complete. An urban environment was chosen because most traffic crashes occur on roads in built‐up areas (Department for Transport, 2016). Prior to driving the trial route, the participants were told to treat the drive as if it were a real drive in the real world. They were told that the speed limit was 30 mph and to drive straight ahead (i.e., not to turn at any junctions). The measure of speeding behaviour used in the data analysis was the percentage of the trial route that the participants spent driving over the 30 mph speed limit. This was operationalized as 30.50 mph or to prevent microfluctuations in speed around 30 mph from unduly influencing the results.

Previous research (McCartan & Elliott, 2018) has shown that measures of speeding behaviour obtained in the present driving simulator correlate well with self‐reported measures of speeding in the real world (*r *=* *.65, *p *<* *.001). The demographic and sociocognitive variables that are typically associated with real‐world speeding behaviour and traffic‐crash rates are also associated with speeding behaviour as measured on this driving simulator. More specifically, accumulated research in road safety has shown that age and driving experience are the key demographic predictors of both real‐world speeding and traffic‐crash risk, with younger and less experienced drivers being found to speed more often and have higher traffic‐crash rates than older and more experienced drivers (e.g., Department for Transport, 2016; McCartt, Mayhew, Braitman, Ferguson, & Simpson, 2009; Stradling *et al*., 2003). Re‐analyses of data from an independent study by Brewster, Elliott, McCartan, McGregor, and Kelly (2016) showed that both these demographic variables were reliable predictors of both mean speed (for age: β = −.27, *p* < .001; for driving experience: β = −.25, *p* < .01) and the proportion of time that participants spend driving over the speed limit (for age: β = −.26, *p* < .01; for driving experience: β = −.23, *p* < .01) on this driving simulator. Conner *et al*. (2007 [study 2]) showed that the sociocognitive variables that predict on‐road vehicle speeds in the real world were behavioural intention (β = −.35, *p* < .01), perceived behavioural control (β = −.03, *p* < .05), and moral norm (β = −.21, *p* < .05). Re‐analysis of the data collected by Brewster *et al*. (2016) showed that behavioural intention (β = −.35, *p* < .01), perceived behavioural control (β = −.14, *p* < .05), and moral norm (β = −.16, *p* < .05) also predicted vehicle speed in this driving simulator.

## Results

### Descriptive statistics and correlations

The sample means, standard deviations, and correlations for the explicit and implicit attitude measures and the measure of speeding behaviour are shown in Table 3. The sample mean for the explicit positive attitude dimension was below the scale midpoint (i.e., 5), which indicates that the participants did not, on average, explicitly evaluate the positive outcomes of exceeding the speed limit very positively. The sample mean for the explicit negative attitude dimension was towards the top end of the scale, indicating that the participants, on average, explicitly evaluated the negative outcomes of exceeding the speed limit as very negative. The sample mean for the implicit positive attitude dimension was below zero, indicating that the participants, on average, did not have strong positive implicit attitudes towards speeding. The sample mean for the implicit negative attitude dimension, on the other hand, was greater than zero, indicating that the participants, on average, had negative implicit attitudes towards speeding. The mean on the measure of behaviour was 26.70, indicating that the participants, on average, exceeded the speed limit for just over a quarter of the simulator drive.

**Table 3:** Descriptive statistics and correlations for all attitude measures and speeding behaviour

Variable	1.	2.	3.	4.	5.	Mean (*SD*)
1. Behaviour	–	.53***	−.30**	.24**	−.07	26.70 (27.10)
2. Explicit positive		–	−.52***	.05	−.03	3.20 (1.86)
3. Explicit negative			–	−.13	.02	7.53 (1.42)
4. Implicit positive				–	.14	−0.16 (0.29)
5. Implicit negative					–	0.22 (0.28)

Note

**p <* *.01; ***p <* *.001.

The correlations in Table 3 show that the positive and negative dimensions of explicit attitudes were negatively correlated, meaning that the more the participants evaluated the positive outcomes of exceeding the speed limit as being positive, the less they evaluated the negative outcomes as being negative. However, the correlation was below *r *=* *.70, which is the conventionally accepted criterion for demonstrating independence amongst constructs (Tabachnick & Fidell, 1996). Similarly, the positive and negative dimensions of implicit attitudes were independent because they were not correlated significantly.

In line with expectations, the positive dimension of explicit attitudes was positively correlated with behaviour (i.e., the more participants evaluated the positive outcomes of exceeding the speed limit as positive, the more they exceeded the speed limit) whereas the negative dimension of explicit attitude was negatively correlated with behaviour. Similarly, the positive dimension of implicit attitudes was positively correlated with behaviour. However, the negative dimension of implicit attitudes was not.

### Predicting behaviour using explicit and implicit measures of bidimensional attitudes

A two‐step hierarchical multiple linear regression was conducted to test both hypotheses 1 and 2 (see Table 4). The dependent variable was the measure of speeding behaviour. The independent variables at step 1 were the explicit positive and negative dimensions of attitude. The implicit positive and negative dimensions of attitude were added to the regression at step 2.

**Table 4:** Hierarchical multiple linear regression predicting speeding behaviour from the explicit and implicit positive and negative dimensions of attitude

Step	Predictor	*R* ^2^	$R_{\mathrm{Change}}^{2}$	*F*_change_	β at step 1	β at step 2
1.	Explicit attitudes
	Positive dimension	.28	.28	23.96***	.51***	.51***
	Negative dimension				−.04	−.01
2.	Implicit attitudes
	Positive dimension	.33	.05	4.628*	–	.22**
	Negative dimension				–	−.09

Note

*p <* *.05; **p <* *.01; ***p <* *.001.

In support of hypothesis 1, Table 4 shows that 28% of the variance in speeding behaviour was account for at step 1 of the regression model. The positive dimension of explicit attitudes was an independent predictor. The negative dimension of explicit attitudes was not. Also in support of hypothesis 1, the positive dimension of explicit attitude had a significantly larger standardized regression coefficient than did the negative dimension, *t*(125) = 5.36, *p *<* *.001.

In support of hypothesis 2, Table 4 also shows that there was a 5% increase to explained variance in speeding behaviour at step 2 of the regression model.^3^ The positive dimension of implicit attitude was an independent predictor. The negative dimension of implicit attitude was not. Also in support of hypothesis 2, the positive dimension of implicit attitude had a significantly larger standardized regression coefficient than did the negative dimension, *t*(123) = 2.71, *p* < .01. The positive dimension of explicit attitudes remained an independent predictor of behaviour at step 2, and the negative dimension of explicit attitudes still did not predict behaviour. Also at step 2, the positive dimension of explicit attitude still had a significantly larger standardized regression coefficient than did the negative dimension of explicit attitude, *t*(123) = 5.13, *p* < .001.^4^

## Discussion

The aim of this research was to test the effects of implicit bidimensional attitudes on drivers’ speeding behaviour over and above the effects of explicit bidimensional attitudes. Hypothesis 1 was that the positive and negative dimensions of explicit attitudes would account for a significant proportion of the variance in behaviour, with the positive dimension being the stronger predictor. Hypothesis 2 was that the positive and negative dimensions of implicit attitudes would account for a significant increment to explained variance in behaviour, with the positive dimension being the stronger predictor.

### Bidimensional effects of explicit and implicit attitudes on behaviour

In support of hypothesis 1, the positive and negative dimensions of explicit attitudes together accounted for a significant proportion of the variance in speeding behaviour. The positive dimension was also found to predict speeding behaviour to a significantly greater extent than the negative dimension. The results from this study therefore extend the findings from studies of unidimensional attitudes (e.g., Armitage & Conner, 2001; Eagly & Chaiken, 1993) in which attitudes are conceptualized as either positive or negative evaluations. They also support the positivity bias that is typically found in previous studies of bidimensional attitudes with the positive attitude dimension being more predictive of behaviour than the negative dimension (Elliott, Brewster, *et al*., 2015; McCartan & Elliott, 2018).

It is worth considering, however, that the findings are not consistent with Lawton *et al*.'s (2007 [study 1]) research on expectancy beliefs about speeding in which beliefs about the likelihood of negative affective outcomes had larger standardized regression weights in the prediction of speeding behaviour than did beliefs about the likelihood of positive affective outcomes. The reason for the discrepancy is unlikely to be a result of Lawton *et al*. (2007) focusing on affective beliefs and the present research focusing on attitudes more generally (i.e., overall measures of cognitive plus affective attitudes). This is because supplementary analyses showed that the positive dimension of attitude still predicted behaviour to a greater extent than the negative dimension when cognitive and affective attitudes were separated (see footnote 3). One possible reason for the discrepant finding is that the mean age of the drivers in this sample was 23 years, and in Lawton *et al*. (2007 [study 1]), it was 49 years. It is possible that the older sample used in Lawton *et al*. (2007 [study 1]) had accumulated more experience of the negative consequences of speeding, thus reinforcing their beliefs about the negative outcomes of this behaviour, which in turn would be expected to increase the relationship with behaviour (cf. Fazio & Zanna, 1981). However, in Elliott, Brewster, *et al*. (2015 [study 3]), the mean age of the sample was 56 years. That study also focused on recent speed limit offenders, all of whom had received a recent negative outcome for their behaviour (being caught by the police in the last 4 months). It was still found that the positive dimension of attitude was more predictive of subsequent speeding than was the negative dimension, in line with the findings of this study and most other studies of outcome beliefs (e.g., Anderson *et al*., 2002; Fromme *et al*., 1997; Lawton *et al*., 2007 [study 2]; Lee *et al*., 1999; Rhodes & Conner, 2010). Although future research might usefully identify the conditions under which the positive dimension of attitude is more predictive of behaviour than the negative dimension (and vice versa), the present findings are consistent with the idea that the positive dimension of attitude is, in general, the primary dictator of behaviour, with individuals’ evaluations of positive behavioural outcomes outweighing their evaluations of negative behavioural outcomes when deciding to act (but see Lawton *et al*., 2007 [study 1]).

It is also worth noting that the negative dimension of explicit attitude was not a significant predictor of behaviour in this study. While it has been shown to predict behaviour in previous research, along with the positive dimension, the effect size has been small. For example, Elliott, Brewster, *et al*. (2015 [study 3]) showed that the beta‐weight for the negative dimension of explicit attitude in the prediction of speeding behaviour on both urban and rural roads was just β = −.11 (compared with β = .31 and β = .38 for the positive attitude dimension on urban and rural roads, respectively). Thus, the evidence, overall, illustrates the utility of the positive attitude dimension in the prediction of behaviour at the expense of the negative attitude dimension.

In support of hypothesis 2, this study demonstrated that the positive and negative dimensions of implicit attitudes together accounted for a significant increment to explained variance in speeding behaviour over and above the variance that was accounted for by the positive and negative dimensions of explicit attitudes. This finding is therefore in line with research on unidimensional attitudes in which implicit measures of attitudes have been found to add variance to behaviour over and above explicit attitudes (e.g., Greenwald *et al*., 2009). The findings imply that spontaneous processes, tapped by implicit attitudes, are important in dictating behaviour along with more deliberative, controlled processes, tapped by explicit attitudes (see Fazio, 1990). Additionally, it is worth noting that the explicit and implicit attitude measures of bidimensional attitudes were uncorrelated in this study, indicating that the measures tapped conceptually different types of attitudes that independently predicted behaviour, consistent with research on unidimensional attitudes (see Perugini, 2005; Wilson, Lindsey, & Schooler, 2000).

Also in support of hypothesis 2, the positive dimension of implicit attitude was a significantly stronger independent predictor of speeding behaviour than was the negative dimension. The results therefore demonstrate, for the first time, that the positivity bias found with explicit bidimensional attitude–behaviour relationships generalize to implicit bidimensional attitude–behaviour relationships. The implication is that behaviour is dictated by evaluations of positive behavioural outcomes at the expense of the negative behavioural outcomes at both the explicit level of cognitive functioning (i.e., when an individual has the motivation and opportunity to think about what action to take) and the implicit level (i.e., when behaviour is more reactive or automatic).

The positivity bias that was found with regard to implicit bidimensional attitudes is particularly important because bidimensional attitudes have been measured in previous research using self‐reported questionnaires, which can be criticized for being susceptible to cognitive (e.g., Murdock, 1962), affective (e.g., Mayer *et al*., 1995), and self‐presentation biases (e.g., Gur & Sackeim, 1979; Paulhus & Reid, 1991). On the other hand, measures of implicit attitudes from IATs are less vulnerable to these criticisms (e.g., Banse *et al*., 2001). The positivity bias found in previous studies of explicit bidimensional attitudes, with the positive dimension predicting speeding behaviour to a significantly greater extent than the negative dimension (e.g., Elliott, Brewster, *et al*., 2015; McCartan & Elliott, 2018), can therefore be held with greater confidence.

### Implications for behaviour‐change interventions

The finding that the positive dimension of explicit attitude was a stronger predictor of speeding behaviour than was the negative dimension has important implications for behaviour‐change interventions (e.g., road safety education). The finding suggests that interventions focusing on explicit attitudes should primarily aim to address the positive outcomes of speeding. For instance, interventions could reinforce the idea that many of the positive outcomes of speeding (e.g., reaching one's destination more quickly) are not likely in many circumstances (e.g., road and traffic conditions often tend to reduce any time savings). Conversely, the finding that the negative attitude dimension did not independently predict speeding suggests that behaviour‐change interventions are likely to be met with limited success if they target only negative outcomes. Indeed, interventions that aim to improve road safety (see Carey, McDermott, & Sarma, 2013; Parker, Stradling, & Manstead, 1996; Stead, Mackintosh, Tagg,  &  Eadie, 2002) and health more generally (see Conner & Norman, 2005) typically focus on the negative outcomes of risky behaviours and the long history of such fear inducing messages having a rather limited effect on behaviour is consistent with the results of this study. More generally, research in road safety has shown that educational interventions that focus on drivers’ attitudes are generally ineffective at modifying driving behaviour and crash risk (Helman, Grayson, & Parkes, 2010; Kinnear *et al*., 2013). Failure to target interventions appropriately is a possible explanation for this finding.

The finding that implicit attitudes predicted behaviour over and above explicit attitudes also has important implications for behaviour‐change interventions. Specifically, the findings imply that interventions are needed to focus on the automatic (implicit) cognitive processes that guide behaviour in addition to more rational (explicit) processes. ‘Evaluative conditioning’ tasks (e.g., Olsen & Fazio, 2001, 2006) are designed to alter implicit associations between behaviours (e.g., speeding) and evaluative attributes (e.g., ‘good’). Such tasks might be useful for changing implicit attitudes towards speeding. In line with the findings of this study, such tasks would need to target implicit associations between speeding and positive, rather than negative, evaluative responses. Alternatively, interventions might usefully prevent implicit attitudes from guiding behaviour in the first place. For example, ‘thought stopping’ tasks (e.g., Foa *et al*., 2005) are known to prevent unwanted cognitions. Such tasks might therefore be able to prevent the positive dimension of implicit attitudes from being activated automatically in response to salient cues associated with speeding and therefore prevent this risky behaviour from being primed. More generally, given that both explicit and implicit attitudes independently predicted speeding in this study, a multipronged intervention approach is recommended through which the positive dimensions of both explicit and implicit attitudes are targeted (e.g., Rydell & McConnell, 2006). Further research is needed to test the sorts of interventions discussed above.

### Methodological considerations

While this study has important implications for both theory and practice, a number of methodological features need to be considered when interpreting the data. First, the sample comprised mainly university students, which resulted in a sample that was younger than the UK driving population (mean age: 22.66 years old vs. 48.8 years old for the UK driving population; DVLA, 2016). However, it is well established that young drivers are overrepresented in traffic crashes (Department for Transport, 2016). Identifying the constructs (e.g., attitudes) that contribute towards the risk‐increasing behaviours of this group (e.g., speeding) is therefore important. It is also worth re‐iterating that the pattern of findings was broadly consistent with previous studies on bidimensional attitudes in which participants were non‐university students (e.g., Elliott, Brewster, *et al*., 2015 [study 3]).

A second methodological feature of this study that needs to be considered when interpreting the results is that behaviour was measured in a driving simulator rather than the real world. As discussed in the Method section, the simulator used in this study has been shown to generate measures of speeding behaviour that significantly correlate with self‐reported measures of real‐world speeding. More importantly, the same constructs that typically predict speeding in the real world (e.g., age, driving experience, behavioural intentions, perceived behavioural control, and moral norm) have been shown to predict speeding on the driving simulator. This supports the findings from other validation studies of driving simulators more generally (e.g., Helman & Reed, 2015). It is also important to note that driving simulators enable the measurement of objective behaviour under full experimental control, in which all participants are exposed to the same environmental conditions. Therefore, they eliminate confounding factors that are often found in the real world (e.g., experience of difficult traffic conditions across participants).

A third methodological feature of the present study that needs to be considered is that implicit attitudes were measured using single‐attribute IATs, in which a single‐attribute category (e.g., ‘good’ or ‘bad’) was paired with either ‘speeding’ or ‘complying’ and participants were asked to categorize target items relating to the three categories. The risk is that the IATs might have been measuring attitudes towards complying with the speed limit rather than attitudes towards exceeding the speed limit. This is potentially problematic because research has demonstrated that ‘doing’ cognitions (i.e., cognitions that relate to performance of a behaviour such as speeding) are separate from ‘not doing’ cognitions (i.e., cognitions that relate to not performing a behaviour such as avoiding speeding, or complying with the speed limit; e.g., Richetin, Conner, & Perugini, 2011). However, attitudes towards exceeding the speed limit and attitudes towards complying with the speed limit are usually found to be highly correlated and therefore typically have the same predictive utility (see Conner *et al*., 2007; Elliott, 2012; Elliott, Armitage, & Baughan, 2003; Letirand & Delhomme, 2005). Furthermore, the IATs developed for this study were shown to be psychometrically reliable and the finding that the positive dimensions of both explicit and implicit attitudes were stronger predictors of behaviour than were the negative dimensions provides evidence of construct validity. Future studies might usefully employ the single‐attribute IATs developed in this study, along with measures of explicit attitudes, to test bidimensional attitude–behaviour relationships with regard to behaviours other than speeding.

A fourth methodological feature of this study is that the measures of explicit attitudes (both dimensions) were based primarily on items that tapped cognitive attitudes rather than affective attitudes whereas the measures of implicit attitudes (both dimensions) were based primarily on attribute items that tapped affective attitudes. This raises the possibility that the implicit attitude measures accounted for additional variance in behaviour over and above the variance accounted for by the explicit attitude measures because affective attitudes are generally stronger predictors. However, it was found that implicit attitudes still accounted for additional variance in speeding behaviour over and above the variance accounted for by explicit attitudes when we separated the cognitive and affective attitude items (see footnote 3). Although this research was not designed to address the distinction between cognitive and affective attitudes, and future research might usefully do so (e.g., by developing separate IATs for cognitive and affective attitudes), the conclusion that implicit bidimensional attitudes increase the prediction of behaviour over and above explicit bidimensional attitudes seems to hold for both cognitive and affective attitudes.

A fifth methodological feature of this study that is worthy of discussion is that the positive dimensions of both explicit and implicit attitudes were marginally more reliable than were the measures of the negative dimensions. This raises the possibility that differential regression attenuation (e.g., Goodwin & Leech, 2006) accounted for the greater prediction of behaviour by the positive attitude dimensions compared with the negative attitude dimensions. However, the positive dimensions of both explicit and implicit attitudes predicted behaviour to a greater extent than did the negative dimensions even when controlling for the differences in measurement reliability (see footnote 4).

A final methodological feature that needs to be considered is that the standard deviations for the positive attitude dimensions were greater than were the standard deviations for the negative attitude dimensions, meaning differences in measurement variability may also have accounted for the greater prediction of behaviour by the positive attitude dimensions. However, the variances of the positive and negative dimensions of implicit attitudes were not statistically significant. While the variance in the positive dimension of explicit attitude was greater than was the variance in the negative dimension, the positive dimension was still a significantly stronger predictor of behaviour even when correcting for the difference (see footnote 4).

### Conclusions

This research shows that the positive attitude dimension is a stronger predictor of behaviour than was the negative dimension and therefore supports the positivity bias found in previous studies of the bidimensional attitude–behaviour relationship (e.g., Elliott, Brewster, *et al*., 2015; McCartan & Elliott, 2018). This result was found for explicit attitudes and, for the first time, implicit attitudes. Attitude‐change interventions might usefully target the positive dimension of both explicit and implicit attitudes. Further research is needed to test the effectiveness of these interventions.

## Funding

The research reported in this article was supported by funding from the Economic and Social Research Council (reference: ES/J500136/1) and the Big Lottery Fund (reference: IID/1/01047171871).

## Footnotes

1 Note that this conceptual redundancy also manifests statistically. For instance, when a measure of attitude ambivalence was calculated in this study, following standard practice (e.g., Thompson *et al*., 1995), 100% of its variance was predicted by the positive and negative attitude dimensions. Analyses testing the moderating effects of attitude ambivalence on the relationships between the positive and negative attitude dimensions, on the one hand, and behaviour, on the other hand, were therefore not only conceptually redundant but also not possible due to perfect collinearity (e.g., Hair, Anderson, Tatham, & Black, 1998).

2 Blocks 2 and 4 are referred to as ‘practice blocks’ in the IAT literature, and blocks 3 and 5 are referred to as ‘test blocks’. While some researchers treat blocks 2 and 4 as genuine practice bocks, Greenwald *et al*. (2003) recommend that they are used to calculate the final IAT measure of attitudes along with blocks 3 and 5 on the basis it leads to larger correlations between implicit and explicit attitude measures than when either the practice or test blocks are used on their own. We therefore followed this accepted procedure. It should be noted, however, that the findings reported in this study were the same regardless of whether or not the ‘practice blocks’ were used to calculate the implicit attitude measures.

3 Research on unidimensional attitudes shows that affective attitudes are typically stronger predictors of behaviour than are cognitive (instrumental) attitudes (e.g., Lawton *et al*., 2009) and that implicitly measured attitudes add little to the prediction of behaviour over and above explicitly measured affective attitudes (e.g., Conner, Prestwich, & Ayres, 2011). This raises the possibility that the additional variance accounted for at step 2 of the regression model was due to the implicit measures containing more affective than cognitive attribute items (i.e., each IAT required the participants to categorize four emotive attributes [e.g., ‘happy’, ‘fun’, ‘wonderful’, and ‘enjoyable’] and just one instrumental attribute [e.g., ‘positive’]) and the explicit measures containing more cognitive than affective attitude items (i.e., the questionnaires required the participants to rate three instrumental items [e.g., ‘positive’, ‘rewarding’ and ‘beneficial’] and just one affective item [e.g., ‘pleasant’]). To rule out this possible explanation for the results, the regression analysis presented in the main text was re‐run with explicit and implicit attitude measures that were computed using just the cognitive attitude items and, again, using just affective attitude items. In both cases, the pattern of results was the same as reported in Table 4.

4 The positive attitude dimensions (both explicit and implicit) had slightly better internal reliabilities and larger standard deviations than did the negative attitude dimensions (see Method section). This raises the possibility that the larger standardized regression coefficients for the positive versus negative attitude dimensions were attributable to differential regression attenuation or differences in measurement variance (e.g., Goodwin & Leech, 2006). To rule out differential regression attenuation, the regression presented in the main text was re‐run using the disattenuated correlation matrix (e.g., Muchinsky, 1996). The pattern of findings was the same as presented in Table 4. To rule out measurement variance, the variance within each attitude measure was calculated. Two repeated measures analyses of variance (ANOVAs) were then conducted testing the difference in variance between the positive and negative implicit attitude dimensions (ANOVA 1) and the positive and negative explicit attitude dimensions (ANOVA 2). ANOVA 1 was not statistically significant, *F*(1, 130) = 0.172, *p *=* *.679. However, ANOVA 2 revealed that the variability in the positive dimension of explicit attitude was greater than was the variability in the negative dimension of explicit attitude, *F*(1, 130) = 15.29, *p* < .001. Given the procedures for estimating regression coefficients while controlling for measurement variability are problematic (Glass & Hopkins, 1996), we therefore removed the 12 cases from the sample that contributed most towards the variability in the explicitly measured positive attitude dimension. A repeated measures ANOVA showed that the difference in the variability between explicitly measured positive and negative attitude dimensions was no longer statistically significant, *F*(1, 118) = 3.50, *p *=* *.064. We then re‐ran the regression presented in the main text with the aforementioned 12 cases removed and the pattern of findings was the same.
